# Anastomotic leakage after esophagogastric resection increases recurrence risk and impairs long-term survival – a propensity score-matched analysis

**DOI:** 10.1186/s12893-026-03681-x

**Published:** 2026-04-10

**Authors:** Rolidy Mariee Jimenez Henriquez, Mikheil Kalandarishvili, Veronika Lummer, Jürgen Weitz, Daniel E. Stange, Felix Merboth

**Affiliations:** 1 Department of Visceral, Thoracic and Vascular Surgery, Faculty of Medicine Carl Gustav Carus, University Hospital, Technische Universität Dresden, Fetscherstrasse 74, Dresden, 01307 Germany; 2 National Center for Tumor Diseases (NCT/UCC), Dresden, Germany: German Cancer Re-search Center (DKFZ), Heidelberg, Germany; University Hospital and Faculty of Medicine Carl Gustav Carus, Technische Universität Dresden, Dresden, Germany; Helmholtz-Zentrum Dres-den-Rossendorf (HZDR), Dresden, Germany; 3 German Cancer Research Center (DKFZ), Heidelberg, Germany; 4 University Hospital and Faculty of Medicine Carl Gustav Carus, Technische Universität Dresden, Dresden, Germany; 5 Helmholtz-Zentrum Dresden-Rossendorf (HZDR), Dresden, Germany

**Keywords:** Anastomotic leakage, Esophagectomy, Gastrectomy, Upper gastrointestinal malignancies, Tumor recurrence, Long-term survival

## Abstract

**Background:**

Esophagogastric resection is the curative treatment for upper gastrointestinal (uGI) tumors but carries risks such as anastomotic leakage (AL). While AL is known to negatively influence oncological outcomes in other cancer entities, its impact on long-term survival in uGI tumors remains unclear. The aim of the study was to determine the rates of tumor recurrence (TR), recurrence patterns, and overall survival (OS) in patients who experienced AL after esophagogastric resection.

**Materials and methods:**

This retrospective cohort study was conducted at the University Hospital Carl Gustav Carus Dresden from January 2013 to December 2019. Patients who underwent esophagectomy or gastrectomy for histologically confirmed carcinoma of the uGI tract were included. After 2:1 propensity score matching, a total of 185 patients with complete 5-year-follow-up were analyzed.

**Results:**

Overall tumor recurrence rates did not differ significantly between patients with AL (AL+) and without AL (AL-) (46.3% vs. 39.0%, *p* = 0.386). However, stratified analysis revealed a higher risk of hematogenous recurrence in the AL+ group (HR 1.701, 95% CI 1.051–2.753, *p* = 0.031) in the cause-specific Cox model, while this association was not significant in the Fine–Gray analysis accounting for competing risks. Median OS was worse in the AL+ group (33 vs. 42 months, *p* = 0.007).

**Conclusion:**

AL after esophagogastric resection was associated with worse long-term OS and a higher current risk of hematogenous recurrence. Since this association was observed only in the cause-specific Cox model and not in the Fine–Gray model, results should be interpreted cautiously. Although overall recurrence rates were similar, the pattern of recurrence differed, highlighting the importance of analyzing recurrence types. Strategies to reduce AL and further research into its biological impact are warranted.

**Supplementary Information:**

The online version contains supplementary material available at 10.1186/s12893-026-03681-x.

## Introduction

Surgical resection, including esophagectomy and gastrectomy, remains a crucial component in the curative management of patients with upper gastrointestinal (uGI) tract malignancies. Complete (R0) resection and adequate lymphadenectomy (D2 or two-field) are the premises for disease free survival. However, oncological uGI surgery is morbidity prone, and anastomotic leakage (AL) is among the most severe and clinically significant postoperative complications [1]. While the immediate risks associated with this complication are well documented, the long-term consequences, particularly its impact on overall survival (OS), recurrence-free survival (RFS) and tumor recurrence (TR) rates, are less clearly understood.

AL has been extensively studied in other gastrointestinal malignancies, such as rectal cancer. A meta-analysis by Karim et al. demonstrated that AL following curative rectal cancer surgery is associated with a reduction in long-term OS [2].

Similarly, emerging evidence suggests that after esophageal resections, AL may increase the risk of local TR, as shown in the systematic review by Aurello et al. [3], and adversely impact OS, as reported by Andreou et al. [4]. Several pathophysiological mechanisms have been proposed to explain the adverse impact of AL on long-term outcomes. For instance, the leakage induces a pronounced local and systemic inflammatory response, characterized by the release of proinflammatory cytokines, which may promote residual tumor cell proliferation and metastatic spread [5, 6]. Furthermore, the occurrence of AL often delays or even precludes the administration of adjuvant therapies, such as chemotherapy or chemoradiotherapy, thereby potentially compromising long-term oncologic outcomes [7].

Despite established correlations in rectal cancer, the specific relationship between AL and oncological outcomes, such as TR and long-term survival, in uGI cancer remains controversial. We hypothesized that postoperative anastomotic leakage after curative esophagogastric resection is associated with reduced overall survival and increased tumor recurrence. This study aimed to test this hypothesis in patients with upper gastrointestinal malignancies.

## Materials and methods

### Study design

This study is a retrospective cohort study conducted at the Department of Visceral, Thoracic, and Vascular Surgery at the University Hospital Carl Gustav Carus, Dresden. The study period spanned from January 2013 to December 2019. It included patients who underwent elective esophagectomy, transhiatal gastrectomy, or total gastrectomy in curative intent for histologically confirmed invasive carcinoma of the uGI tract, with or without prior neoadjuvant treatment; open, laparoscopic/thoracoscopic or robot-assisted procedures were included. Depending on the localization of the tumor, esophageal resections were performed either as an Ivor Lewis procedure with intrathoracic anastomosis or as a McKeown procedure with cervical anastomosis, both with gastric tube conduit reconstruction and a two-field lymphadenectomy. In transhiatal and total gastrectomy, a D2 lymphadenectomy and reconstruction as an end-to-side esophagojejunostomy were performed. In most cases, the anastomosis was created using a circular stapler; only in minimally invasive procedures were side-to-side anastomoses occasionally created using a linear stapler.

The exclusion criteria included: patients who had additional malignancies, underwent palliative surgery, endoscopic mucosal resection or endoscopic submucosal dissection. The observation period was 5 years post-surgery. The study protocol was reviewed by the local ethics committee (BO-EK-38012021) and was conducted in accordance with the Declaration of Helsinki and its subsequent amendments. A waiver was granted for informed consent.

### Follow-up

All patients who underwent esophagogastric resection for uGI cancer were followed-up according to the guidelines of the local Comprehensive Cancer Center (National Center for Tumor Diseases), which specified the timing and type of postoperative examinations. These examinations included clinical visits, imaging procedures (such as computed tomography [CT] scans or endoscopies), and laboratory tests, conducted every 3 to 4 months initially in the first year, then biannually. The follow-up monitored recurrence, complications, and assessed overall recovery.

### Definition of AL and TR

AL was defined according to the Esophagectomy Complications Consensus Group (ECCG) as a “full-thickness GI defect involving the esophagus, anastomosis, staple line, or gastric conduit, irrespective of the clinical presentation or method of detection, which required reintervention or reoperation” [8]. In our cohort, all AL were initially confirmed radiologically by the presence of extraluminal contrast on CT. Subsequently, all cases were endoscopically verified as full-thickness defects. Mucosal necrosis or ulcerations without transmural involvement were not considered as leakage.

TR was defined as locoregional or distant disease after curative resection and was assessed during routine follow-up using CT and/or PET-CT, endoscopy when indicated, and histological confirmation when feasible. Recurrence was diagnosed based on radiological and/or histological findings and confirmed by multidisciplinary tumor board consensus.

### Study endpoints

The primary objective of the present study was to assess whether AL impacted the TR. The TR rates given are absolute rates after the specified follow-up period of 5 years postoperatively. The secondary objective was to evaluate the OS.

### Statistical analysis

Statistical analyses were performed using the Statistical Package for the Social Sciences software (SPSS, version 28.0; IBM Corp., Armonk, NY, USA) and Python (version 3.12.1; Python Software Foundation, Beaverton, USA). Variables are summarized using median with interquartile range (IQR) and frequencies. To prevent immortal time bias, a time-dependent Cox model was fitted for OS and RFS. Recurrence subtypes were examined using a time-dependent cause-specific Cox model.

Further analyses were performed by landmark analysis starting at postoperative day 30. Patients were divided into two groups based on the presence or absence of AL at this timepoint. To ensure better comparability between the groups, 2:1 propensity score matching without replacement was performed. The following variables were used to calculate the propensity score using the regression models: sex, age, body mass index (BMI), American Society of Anesthesiologists Classification (ASA), histology, neoadjuvant treatment, adjuvant treatment, pathologic Union internationale contre le cancer (UICC) stage, surgical procedure, and surgical approach. Missing data were handled as an additional category. Subsequently, the nearest neighbor method with a caliber on logit width of 0.1 was used to find matching pairs. Standardized mean differences (SMD) were used for the balance metrics and the corresponding Love Plot and Common Support was created (Supplementary Fig. 1).

A p-value of < 0.05 was considered statistically significant. Clinicopathological characteristics of the two groups were compared using Mann-Whitney U test for continuous variables and chi-square (χ²) or Fisher´s exact test for discrete variables. TR rates and 1- to 5-year survival rates were evaluated using Fisher’s exact test and odds/hazard ratios were determined. The OS und the event-free survival for hematogenous recurrences were analyzed with Cox-regression and the log-rank-test and were represented as Kaplan-Meier curves. Recurrence endpoints were analyzed using both Fine–Gray subdistribution hazard models to account for competing risks and cause-specific Cox proportional hazards models. Multiplicity control was performed via Benjamini-Hochberg-correction.

## Results

A total of 466 patients underwent esophagogastric resections at the University Hospital Carl Gustav Carus, Dresden between January 2013 and December 2019 for various indications. Of these, 101 patients were deselected according to the exclusion criteria. From the remaining 349 patients, 52 were excluded due to the lack of follow-up data, primarily due to residence abroad or relocation without updated contact information, with no evidence of systematic bias. The final cohort included 313 patients, who were divided into two groups based on the presence (AL+) or absence (AL–) of AL.

Before propensity score matching, both groups (AL−: *n* = 239; AL+: *n* = 74) showed no significant differences in various baseline parameters such as age, BMI, ASA classification, and risk factors including nicotine or alcohol consumption and diabetes mellitus. However, significant differences were found in oncological relevant factors: patients in the AL+ group received neoadjuvant therapy more frequently (78.4% vs. 66.1%, *p* = 0.007), especially radiochemotherapy (50.0% vs. 28.5%), and were more likely to present with squamous cell carcinoma (39.2% vs. 20.5%, *p* = 0.004) than the AL– group. Moreover, abdominothoracic esophagectomy was significantly more common in the AL+ group (82.4% vs. 56.1%, *p* < 0.001). For detailed information, see Supplementary Table 1.

The median time to diagnosis of AL was 9.0 days (IQR 7.0-11.8), with all leaks detected within the first 30 days postoperatively. The severity was classified according to the ECCG classification: ECCG 1: 18.9%, ECCG 2: 51.4%, and ECCG 3: 29.7%. AL was treated conservatively in 18.9% of cases with a zero diet, in 8.1% with fibrin glue, in 43.2% with endoluminal sponge therapy, in 8.1% with reoperation and re-creation of the anastomosis, and in 21.6% with esophageal diversion. In the entire cohort of 313 patients, the 30-day mortality was 4.5% and the 90-day mortality was 7.7%. The 30-day mortality rate was 6.8% in the AL+ group and 3.8% in the AL- group (*p* = 0.332), while the 90-day mortality rate was 9.5% and 7.1% (*p* = 0.465), respectively.

### Baseline characteristics

After 2:1 propensity score matching, the AL+ group included 67 patients and the AL− group 118 patients. No significant differences were present in demographic or clinical baseline characteristics between the two groups (Table 1). The median age was 63.3 years (± 9.2) in the AL+ group and 64.0 years (± 11.3) in the AL− group (*p* = 0.652). Male patients constituted the majority in both groups: 82.1% in the AL+ group and 79.7% in the AL− group (*p* = 0.853). The mean BMI was 25.6 kg/m² (± 4.2) in the AL+ group and 25.7 kg/m² (± 3.7) in the AL− group (*p* = 0.810). ASA classification was comparable between the groups, with ASA III being the most frequent score in both cohorts. Risk factors for malignancies of the uGI tract—such as chronic nicotine use and alcohol consumption—also showed no significant differences: 41.8% in the AL+ group and 33.1% in the AL− group reported chronic nicotine use (*p* = 0.237). Chronic alcohol consumption was reported in 19.4% vs. 17.8% of patients (*p* = 0.788), and diabetes mellitus was present in 29.9% vs. 22.9% of patients (*p* = 0.298).

**Table 1:** Data on demographical and histopathologic characteristics and perioperative therapy

	AL- (*n* = 118)	AL+ (*n* = 67)	*p*-value	SMD
Age [years]	64.0* (± 11.3)	63.3* (± 9.2)	0.652^	0.071
Sex			0.835	0.062
Female	24 (20.3)	12 (17.9)		
Male	94 (79.7)	55 (82.1)		
BMI [kg/m²]	25.7* (± 3.7)	25.6* (± 4.2)	0.810^	0.036
Smoking	39 (33.1)	28 (41.8)	0.237	0.180
Alcohol	21 (17.8)	13 (19.4)	0.788	0.041
Diabetes mellitus	27 (22.9)	20 (29.9)	0.298	0.158
ASA			0.750	0.073
2	50 (42.4)	26 (38.8)		
3	68 (57.6)	41 (61.2)		
Histology			0.635	0.145
Adenocarcinoma	69 (58.5)	35 (52.2)		
Squamous cell carcinoma	41 (34.7)	28 (41.8)		
Other	8 (6.8)	4 (6.0)		
Resection status			0.654	0.01
R0	109 (92.4)	60 (89.6)		
R1	5 (4.2)	5 (7.5)		
Rx	4 (3.4)	2 (3.0)		
UICC Stage			0.865	0.026
I	31 (26.3)	16 (24.6)		
II	15 (12.7)	17 (26.2)		
III	57 (48.3)	21 (32.3)		
IV	15 (12.7)	11 (16.9)		
Neoadjuvant treatment			0.629	0.148
None	25 (21.2)	12 (17.9)		
Chemotherapy	40 (33.9)	20 (29.9)		
Radiochemotherapy	53 (44.9)	35 (52.2)		
Adjuvant treatment			0.825	0.071
None	103 (87.3)	60 (89.6)		
Chemotherapy	15 (12.7)	7 (10.4)		
Surgical resection			0.028	0.397
Esophagectomy	84 (71.2)	58 (86.6)		
Gastrectomy	34 (28.8)	9 (13.4)		
Surgical approach			0.643	0.144
Open	74 (62.7)	40 (59.7)		
Hybrid	12 (10.2)	5 (7.5)		
MIS	32 (27.1)	22 (32.8)		

n (%), *median (IQR), Chi square test, ^Mann Whitney U test

*AL *anastomotic leakage, *ASA *American Society of Anesthesiologists, *BMI *body mass index, *IQR *interquartile range, *MIS *minimally invasive surgery, *SMD *standardized mean difference, *UICC *Union for International Cancer Control

### Perioperative therapy and tumor histology

Neoadjuvant therapy was administered to 82.1% of patients in the AL+ group and to 78.8% patients in the AL− group (*p* = 0.629). Among them, in AL− group 33.9% received chemotherapy and 44.9% received radiochemotherapy, while in the AL+ group 29.9% received chemotherapy and 52.2% radiochemotherapy. In most cases, neoadjuvant radiotherapy was carried out with 40.0 Gy. Cisplatin-based regimens such as ECF/ECX were most frequently used (47.7% vs. 37.3%), followed by oxaliplatin-based regimens such as FLOT/FLO (10.8% vs. 20.3%) and carboplatin/paclitaxel (12.3% vs. 11.9%). No differences were found between the two groups with regard to the chemotherapy regimens (*p* = 0.453).

Adjuvant therapy was less frequent but not statistically significantly different, with 10.4% in the AL+ group and 12.7% in the AL− group (*p* = 0.825). Both cisplatin-based regimens (4.6% vs. 1.7%) and oxaliplatin-based regimens (1.5% vs. 7.6%) were administered adjuvantly (*p* = 0.248). Adjuvant therapy was started after a median of 8 weeks (IQR 7–12 weeks) in the AL+ group and after 6 weeks (IQR 5–7 weeks) in the AL- group, which did not result in a significant difference (*p* = 0.076). A dose reduction was necessary in 16.7% of patients in the AL+ group and in 25.0% in the AL- group (*p* = 1.000).

Regarding tumor histology, 58.5% in the AL− group were diagnosed with adenocarcinoma and 34.7% with squamous cell carcinoma. In the AL+ group, 52.2% had adenocarcinoma and 41.8% squamous cell carcinoma. Rare histologic entities (e.g., leiomyosarcoma, neuroendocrine tumor) were observed in both groups but were uncommon. No statistically significant differences were found for histology (*p* = 0.635, Table 1).

### Operative characteristics

In the AL− group, 71.2% underwent esophagectomy and 28.8% gastrectomy. The proportion of esophagectomy was higher in the AL+ group, 86.6%, while gastrectomy was less frequent with 13.4%. Even after matching for this variable, a significant difference remained (*p* = 0.028). With regard to the surgical approach, 62.7% in the AL− group underwent conventional open surgery, while 27.1% were treated using minimally invasive or robot-assisted techniques. In the AL+ group, 59.7% had open surgery, and 32.8% were treated minimally invasively (*p* = 0.643, Table 1).

### Overall and recurrence-free survival prior to PSM

**Fig. 1:**
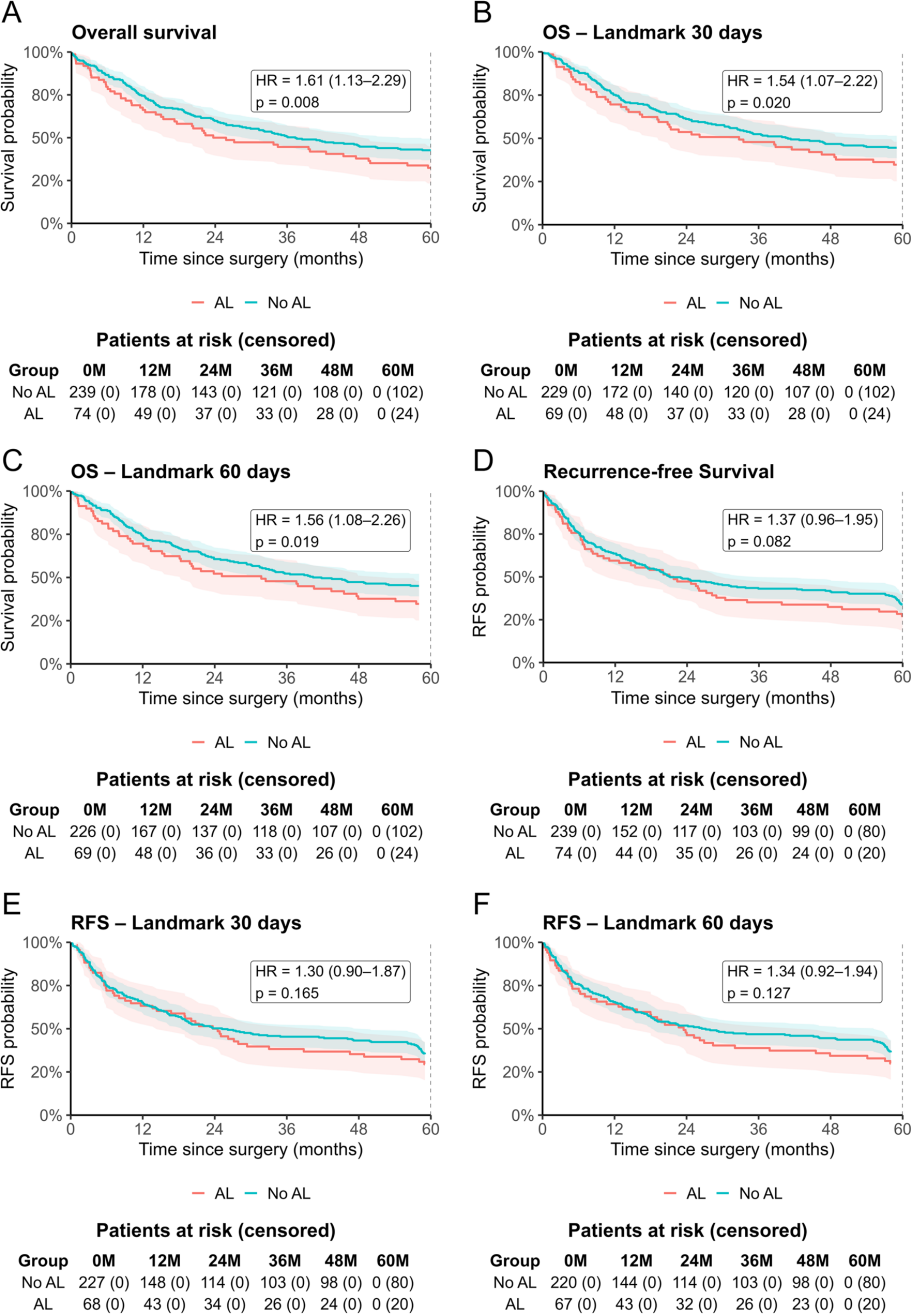
Kaplan-Meier Curves for overall survival and recurrence-free survival of the overall cohort. OS and RFS with AL as time-varying variable. Sensitivity landmark analyses were performed for OS at baseline (**A**), 30 days (**B**) and 60 days (**C**) and for RFS at baseline (**D**), 30 days (**E**) and 60 days (**F**)

In the overall cohort, OS was significantly worse in the AL+ group for both baseline (HR 1.61, 95%CI 1.13–2.29, *p* = 0.008) and landmark analyses at 30 days (HR 1.54, 95%CI 1.07–2.22, *p* = 0.020) and 60 days (HR 1.56, 95%CI 1.08–2.26, *p* = 0.019; Fig. 1A-C). In contrast, RFS did not differ between the two groups at baseline (HR 1.37, 95%CI 0.96–1.95, *p* = 0.082) or at 30 days (HR 1.30, 95%CI 0.90-0.87, *p* = 0.165) or 60 days (HR 1.34, 95%CI 0.92–1.94, *p* = 0.127; Fig. 1D-F).

Histological confirmation of recurrence was performed in 58.3% of patients. In all other cases of suspected recurrence based on imaging, confirmation was provided by a multidisciplinary tumor board.

The OS was better in ECCG 2 compared with the other two severity grades (*p* = 0.039), but the RFS did not differ (*p* = 0.221, Supplementary Fig. 2). Overall, however, the group sizes were very small, so these results must be interpreted with caution.

### Tumor recurrence rates and overall survival after PSM

**Fig. 2:**
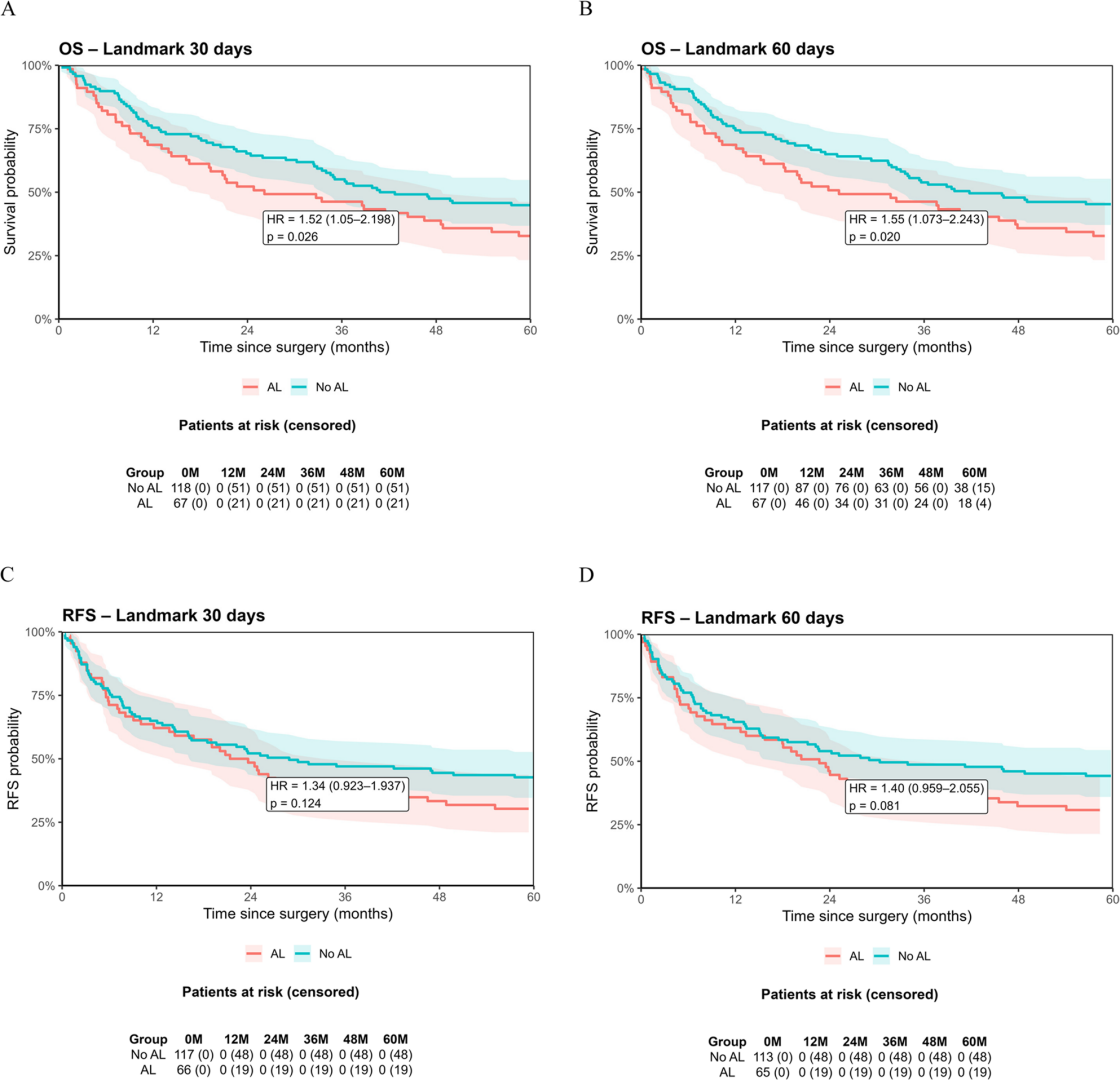
Cumulative incidence functions (CIFs) for overall and hematogenous recurrence. Cumulative incidence of overall and haematogenous recurrence in patients with versus without anastomotic leakage (AL), using a landmark analysis at postoperative day 30. Death was treated as a competing risk, and subdistribution hazard ratios (sHR) were estimated using Fine–Gray models

After PSM the overall recurrence rate did not differ in both groups (46.3% vs. 39.0%, *p* = 0.386; Table 2). Interestingly, hematogenous recurrences were more common in the AL+ group; 37.3% compared to 25.4% in the AL− group but without significance according to the Fine-Grey model (*p* = 0.086, Fig. 2). However, in cause-specific Cox regression, in which competing events are censored, there is a significantly increased risk of hematogenous recurrence in the AL+ group (HR 1.701, 95%CI 1.051–2.753, *p* = 0.031). Among the hematogenous recurrences, pulmonary metastases (52.0% vs. 51.6%) were the most common, followed by hepatic (28.0% vs. 25.8%) and bone (28.0% vs. 22.6%).

**Table 2:** Type and rate of recurrence

Recurrence (overall)	AL- (*n* = 118)	AL+ (*n* = 67)	*p*-value (FG)	HR	95% CI	*p*-value (CSC)
	46 (39.0)	31 (46.3)	0.386	1.309	0.898–1.906	0.160
Lymphatic	20 (16.9)	14 (20.9)	0.486	1.409	0.719–2.759	0.317
regional	12 (10.2)	7 (10.4)	0.977	1.652	0.707–3.864	0.247
distant	12 (10.2)	10 (14.9)	0.327	1.159	0.420–3.198	0.775
Hematogenous	30 (25.4)	25 (37.3)	0.086	1.701	1.051–2.753	0.031
Peritoneal	17 (14.4)	6 (9.0)	0.298	0.655	0.251–1.714	0.389
Local	10 (8.5)	5 (7.5)	0.825	0.976	0.308–3.089	0.967

n (%), Fine-Grey model, cause-specific Cox regression

*AL *anastomotic leakage, *CI *confidence interval, *HR *hazard ratio, *CSC *cause-specific Cox

**Fig. 3:**
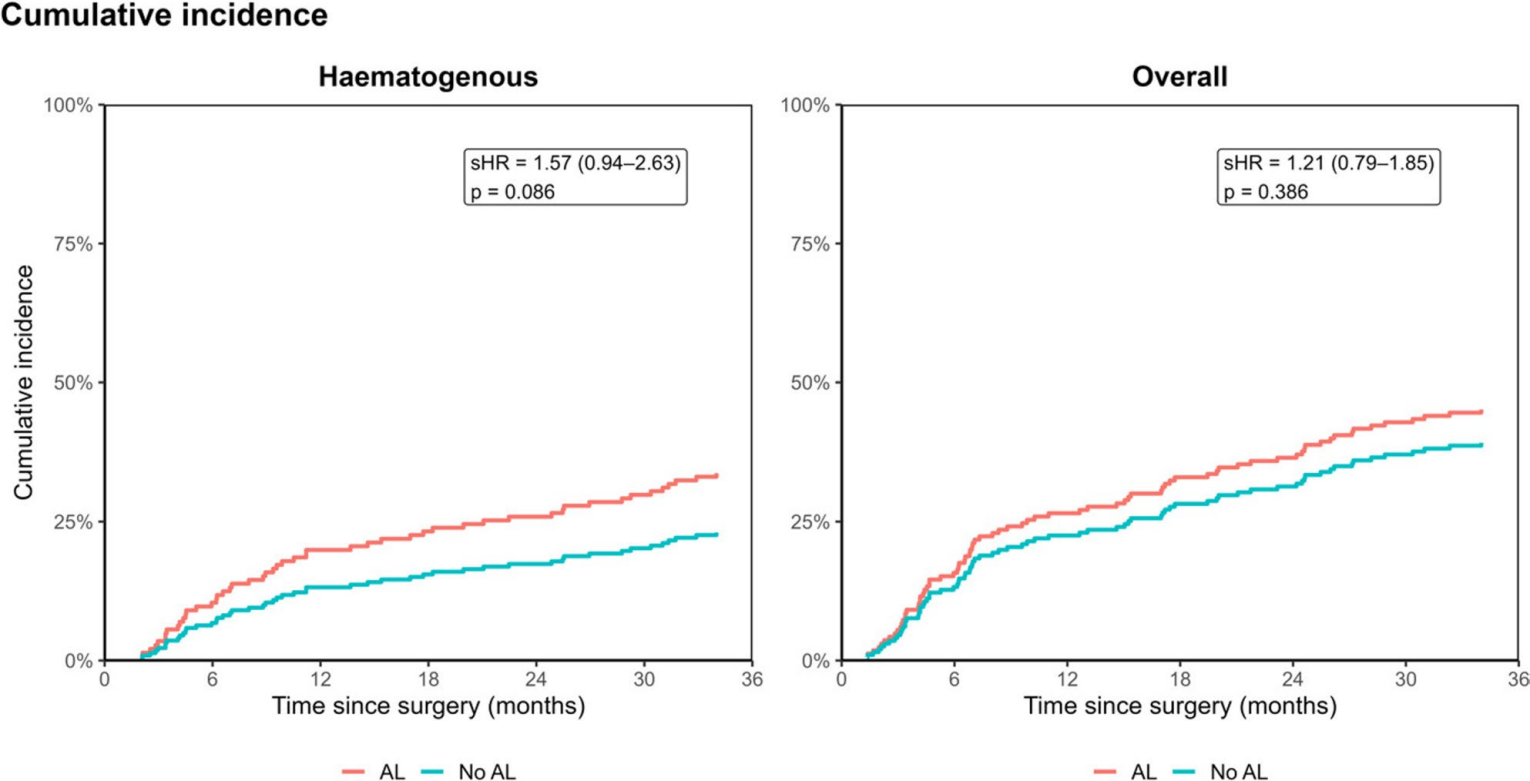
Kaplan-Meier Curves for overall survival and recurrence-free survival of the matched cohort. OS and RFS after propensity score matching. Sensitivity landmark analyses were performed for OS at 30 days (**A**) and 60 days (**B**) and for RFS at 30 days (**C**) and 60 days (**D**)

Lymphatic recurrence occurred in 20.9% in the AL+ group and 16.9% in the AL− group (*p* = 0.486). Regional lymphatic recurrence predominated in both groups (AL+: 10.4%; AL−: 10.2%, *p* = 0.977) compared to distant lymphatic recurrence (AL+: 14.9%; AL−: 10.2%, *p* = 159). Peritoneal carcinomatosis recurrence was observed in 9.0% of the patients in AL+ group and in 14.4% in AL- group (*p* = 0.298). Local TR was detected in 7.5% in the AL+ group and in 8.5% in the AL− group (*p* = 0.976). The median time to first recurrence did not differ significantly (AL+: 11.0, IQR 5.4–23.2 months; AL−: 7.8, IQR 4.2–17.1 months; *p* = 0.285). Patients with AL had a median OS of 33 months (IQR: 10.5–60 months), whereas patients without AL had a median OS of 42 months (IQR: 12.75–60 months, HR 1.844, 95%CI 1.183–2.872, *p* = 0.007, Table 3). The poorer OS was also reflected in the landmark analyses at 30 days (HR 1.519, 95%CI 1.050–2.198, *p* = 0.027) and 60 days (HR 1.551, 95%CI 1.073–2.243, *p* = 0.020; Fig. 3A-B). However, the RFS showed no significant difference in the landmark analyses at 30 days (HR 1.337, 95%CI 0.923–1.937, *p* = 0.124) and 60 days (HR 1.404, 95%CI 0.959–2.055, *p* = 0.081; Fig. 3C-D).

**Table 3:** Overall Survival and 1- to 5-year survival rates

Overall survival [M] (%)	AL- (*n* = 118)	AL+ (*n* = 67)	*p*-value	HR	95% CI
	42* (12.75–60)	33* (10.5–60)	0.007^	1.844	1.183–2.872
1 year	89 (75.4)	46 (68.7)	0.389	1.745	0.936–3.251
2 years	77 (65.3)	35 (52.2)	0.088	2.006	1.186–3.394
3 years	65 (55.1)	31 (46.3)	0.285	1.776	1.092–2.890
4 years	56 (47.5)	26 (38.8)	0.283	1.777	1.123–2.812
5 years	53 (44.9)	22 (32.8)	0.121	1.844	1.183–2.872

n (%), Fisher´s Exact test, *median (IQR), ^Cox regression

*AL *Anastomotic leakage, *CI *confidence interval, *HR* hazard ratio, *IQR *interquartile range, *M *month

Recurrence rates and OS for the overall cohort can be found in Supplementary Tables 2 and 3.

A subgroup analysis for esophagectomies only ultimately showed similar results: poorer OS (HR 2.60, 95%CI: 1.52–4.46, *p* < 0.001, Supplementary Fig. 3), with the risk of hematogenous recurrence only tending to be higher (HR 1.618, 95%CI: 0.925–2.830, *p* = 0.092, Supplementary Table 4). The subgroup analysis also showed that the impact of AL on survival was most significant in patients with squamous cell carcinoma (HR 2,191, 95%CI: 1.092–4.396, *p* = 0.027) and those who had undergone neoadjuvant therapy (HR 1,633, 95%CI: 1.063–2.509, *p* = 0.025) (Supplementary Table 5).

## Discussion

Surgical resection is the standard treatment for patients with uGI cancer, requiring the creation of anastomoses to restore continuity. Despite the increasing use of minimally invasive procedures, the AL rate does not fall below 8%, even at highly specialized centers [9]. Therefore, AL remains a serious and life-threatening complication after esophagogastric resection, with mortality rates of up to 35% after esophagectomy and 18% after gastrectomy [10–13]. Novel and primarily interventional therapy modalities, such as CT-guided drainage systems or endoluminal sponge therapy, have drastically reduced both reoperation rates and mortality in patients with AL [14]. While the immediate effects of AL are therefore better managed, the long-term consequences of AL on TR and survival remain poorly understood, and definitive conclusions cannot be drawn.

After resection of rectal cancer, patients with AL have an increased risk of local TR and decreased long-term OS [2]. These findings are further supported by a comprehensive systematic review and meta-analysis by Yang et al., which included over 38,000 patients [15]. The study demonstrated that AL was associated with a significant reduction in OS after rectal cancer surgery (HR = 1.64; 95% CI: 1.37–1.95; *p* < 0.001). Additionally, patients with AL had a significantly higher risk of local TR (HR = 1.93; 95% CI: 1.57–2.38; *p* < 0.001). Interestingly, no statistically significant association was found between AL and distant metastasis (HR = 1.25; 95% CI: 0.95–1.65; *p* = 0.12), suggesting that the adverse impact of AL in rectal cancer may be primarily limited to promoting local recurrence. One potential biological explanation is that AL may induce prolonged inflammatory reactions within the surrounding tissue, potentially creating a local pro-carcinogenic microenvironment.

In contrast, following esophagogastric resection, our data showed no increase in local TR. A reduced OS could be documented. Although overall tumor recurrence rates were similar between AL + and AL- groups, the pattern of recurrence differed. In particular, hematogenous recurrences were more frequent in the AL+ group, whereas local and lymphatic recurrences did not differ. This suggest that AL may specifically influence the pattern of metastatic spread rather than overall recurrence frequency. Although this difference was not significant in the Fine-Grey model, the cause-specific Cox model showed a significant increase in risk for the occurrence of hematogenous recurrence in the AL+ group with an HR of 1.7 (95% CI 1.051–2.753, *p* = 0.031). The Fine-Grey model compares the absolute risk and includes competing events such as death from other recurrences. The cause-specific Cox model, on the other hand, shows the current risk for a specific event, whereby competing events are censored. This allows the etiology behind the increased risk to be better understood. In our work, the current risk of developing a hematogenous recurrence was higher in the AL+ group, but this difference was not reflected in a different cumulative incidence, probably because competing events balanced out or overlapped the effect over time. This was probably also reflected in the fact that there was no significant difference in RFS.

In line with our data, several large-scale studies demonstrated a negative impact of AL on oncologic outcomes after esophagogastric resection. Andreou et al. identified AL as an independent predictor of both OS and RFS, with hematogenous spread—particularly to the liver—being the predominant recurrence pattern, suggesting a possible systemic effect of AL [4]. Similarly, Kofoed et al. found that AL was significantly associated with increased recurrence risk (HR = 1.63) and all-cause mortality (HR = 1.57), along with a lower 5-year RFS (27% vs. 39%) [16]. Markar et al., in a large multicenter cohort, reported that severe esophageal AL independently predicted worse OS (HR = 1.28) and was associated with significantly increased rates of overall, locoregional, and mixed recurrence patterns [17]. These findings are in line with our observation of a higher rate of hematogenous metastases and a reduced OS in patients with AL.

Aoyama et al. further reported that patients with AL had a significantly shorter OS (35.8 vs. 54.8 months, *p* = 0.022), and AL remained an independent prognostic factor in both univariate and multivariate analyses. Although the underlying physiological mechanism behind this association remains unclear, the authors speculated that an impaired immune response to residual tumor burden may contribute [18]. In a large international multicenter study by Fransen et al., involving 915 patients undergoing minimally invasive esophagectomy, AL was significantly associated with worse OS (*p* = 0.025) [19]. Belmouhand et al. described a non-significant trend toward increased local recurrence in patients with AL and found younger age to be associated with higher recurrence risk and poorer prognosis [20]. However, AL itself was not linked to reduced OS in their analysis; instead, male sex and lymph node positivity were the strongest predictors of worse outcomes.

In contrast to studies highlighting a detrimental impact of AL on oncological outcomes, several investigations have reported more nuanced findings or no impact. For instance, Hii et al. found no significant effect of postoperative complications, including AL, on disease-specific survival [21]. Similarly, Lerut et al. observed that recurrence correlated with concurrent medical and surgical complications, but not with surgical complications alone, such as AL [22]. Tam et al. reported that while postoperative infections were associated with worse OS, AL was not identified as an independent risk factor after adjusting for potential confounding variables [23]. Sugimura et al. also found that although general postoperative complications were associated with significantly worse OS (HR = 2.06; *p* = 0.017), AL alone did not show a statistically significant impact (HR = 1.37; *p* = 0.377) [24]. Supporting this, Kamarajah et al., who stratified patients by AL severity using the Clavien–Dindo Score, reported no significant difference in OS among patients with severe, non-severe, or no AL (*p* = 0.8), with median survival times of 61, 55, and 41 months, respectively [25]. Finally, Kataoka et al. demonstrated that pneumonia was associated with decreased OS, whereas survival was similar between patients with and without AL [26]. These apparently contradictory results may be influenced by variations in study design, patient selection, definitions of complications, and follow-up duration. Notably, among the more recent studies, only one employed a matching methodology to balance baseline characteristics between groups to approximate randomization.

While the biological mechanisms underlying the observed association between AL and hematogenous metastasis can only be addressed using sophisticated model systems, a potential hypothesis is that AL induces a systemic inflammatory state. The systemic inflammatory response to AL may subsequently lead to temporary immunosuppression. For esophagogastric adenocarcinoma, this relationship is increasingly recognized, independent of postoperative complications. Esophagectomy, in and of itself, regardless of infectious or inflammatory complications, appears to potentially exert a stronger influence on the postoperative inflammatory cascade compared to other oncologic procedures [5, 6]. In addition, several studies have demonstrated that baseline upregulation of tumor-associated inflammatory genes and elevated inflammatory markers may be predictive of worse outcomes [27, 28]. AL could further amplify an already heightened inflammatory predisposition. The release of proinflammatory cytokines, such as interleukin-32 and tumor necrosis factor-alpha, contributes to systemic effects and may create a pro-tumorigenic microenvironment in patients with esophageal cancer, including in distant organs [29, 30]. This microenvironment could potentially promote angiogenesis, tissue remodeling, and the survival and proliferation of residual tumor cells that subsequently spread throughout the body—mechanisms that have previously been identified as relevant in cancer progression.

A second frequently cited explanation for poorer OS and more frequent recurrences following AL is the non-administration or delayed administration of adjuvant therapy. A recent meta-analysis showed that delays as short as 4 weeks may increase mortality across various common cancer types, with longer delays potentially further exacerbating outcomes [7]. However, cancers of the uGI tract were not included in this analysis, and the relevance of these results for esophageal and gastric cancer therefore remains uncertain. Moreover, some studies suggest that delayed adjuvant therapy does not always lead to worse survival outcomes [31]. In our cohort, the delay in adjuvant therapy in the AL+ group was only 2 weeks (from 6 to 8 weeks), which did not appear to result in a significant difference. To further address this issue, we included patient and pathological characteristics in our matching process, along with oncological factors such as neoadjuvant and adjuvant therapy, to enhance comparability between the groups. Only after achieving group comparability through matching clear differences emerged, particularly regarding OS and hematogenous recurrences. These results underline the importance of appropriate methodological approaches, especially in studies where randomization is not feasible.

A major limitation of this study is its retrospective, single-center design, which carries inherent risks of selection bias and limits generalizability. To enhance comparability, a 2:1 propensity score matching was performed; nevertheless, residual imbalances in surgical procedures and tumor location may persist and could influence both the risk of anastomotic leakage and recurrence patterns. Further stratification was not feasible due to the relatively small sample size.

The study pooled patients with esophageal squamous cell carcinoma, esophageal adenocarcinoma, and gastric adenocarcinoma, which differ biologically in recurrence patterns, immune profiles, and response to therapy. While our data suggest a potential link between AL and hematogenous recurrence, this mechanism remains speculative and may not act uniformly across all tumor types. Subgroup analyses were limited by sample size and interaction tests were non-significant.

Another limitation arises from the long time frame of the analyzed data, during which perioperative therapy was predominantly cisplatin-based regimens such as ECF or ECX, which no longer reflect current standards of care. Despite these limitations, a key strength of the study is the availability of complete 5-year follow-up data for 313 patients, which allowed robust assessment of long-term outcomes.

In summary, despite its retrospective design, small sample size, and biological and surgical heterogeneity, the study highlights a negative association between AL and oncologic outcomes. Matching and complete follow-up increase confidence in the findings, while cautious interpretation and further research into underlying mechanisms remain warranted.

## Conclusion

Our findings indicate that the 5-year OS of patients with AL was markedly reduced. While overall recurrence rates were similar, the pattern of recurrence differed, with an increased current risk of hematogenous metastases observed in the cause-specific Cox model (HR 1.7), but not in the Fine–Gray analysis. These results underscore the potential negative prognostic effect of AL while highlighting the need for cautious interpretation. They also emphasize the importance of minimizing AL, implementing enhanced follow-up for affected patients, and conducting further research to elucidate the mechanisms linking AL to hematogenous recurrences. 

Our findings further support the need for strategies to reduce the incidence of AL in patients undergoing surgery for uGI tumors. Enhanced monitoring and management approaches for patients who experience and survive AL should be considered. Routine follow-up should include comprehensive imaging, and follow-up intervals should not be extended prematurely. Furthermore, while our study provides insight into the association between AL and oncological outcomes, it also underscores the need for ongoing research in this area. Future studies should focus on clarifying the mechanisms underlying the relationship between AL and hematogenous recurrences following esophagogastric resection. 

## Supplementary Information

Supplementary Material 1.

## Data Availability

The data are available from the corresponding author on request.

## Abbreviations

• AL: Anastomotic Leakage
• CSC: Cause-specific Cox
• CT: Computed tomography
• ECCG: Esophagectomy Complications Consensus Group
• FG: Fine-Grey
• OS: Overall Survival
• RFS: Recurrence-free Survival
• SMD: Standardized mean differences
• TR: Tumor Recurrence
• uGI: upper Gastrointestinal
