# Supplementary material for: Anastomotic leakage after esophagogastric resection increases recurrence risk and impairs long-term survival – a propensity score-matched analysis
Source: BMC Surg. 2026 Apr 10;26:279. doi: 10.1186/s12893-026-03681-x (PMC13088859; doi:10.1186/s12893-026-03681-x)

# Supplementary

# Tables

**Supplementary Table 1**. Data on demographical and histopathologic characteristics and perioperative therapy of overall cohort

|  | **AL- (n=239)** | **AL+ (n=74)** | **p-value** | **SMD** |
| --- | --- | --- | --- | --- |
| **Age** [years] | 64.78 (10.78) | 64.27 (9.55) | 0.715 | 0.050 |
| **Sex** |  |  | 0.979 | 0.026 |
| Female | 46 (19.2) | 15 (20.3) |  |  |
| Male | 193 (80.8) | 59 (79.7) |  |  |
| **BMI** [kg/m²] | 25.24 (4.01) | 25.92 (4.80) | 0.222 | 0.155 |
| **Smoking** | 0.35 (0.48) | 0.39 (0.49) | 0.528 | 0.083 |
| **Alcohol** | 0.18 (0.38) | 0.19 (0.39) | 0.793 | 0.035 |
| **Diabetes mellitus** | 0.26 (0.44) | 0.28 (0.45) | 0.733 | 0.045 |
| **ASA** |  |  | 0.381 | 0.280 |
| 1 | 2 (0.8) | 0 (0.0) |  |  |
| 2 | 86 (36.0) | 31 (41.9) |  |  |
| 3 | 145 (60.7) | 43 (58.1) |  |  |
| 4 | 6 (2.5) | 0 (0.0) |  |  |
| **Histology** |  |  | 0.004 | 0.432 |
| Adenocarcinoma | 176 (73.6) | 40 (54.1) |  |  |
| Squamous cell carcinoma | 49 (20.5) | 29 (39.2) |  |  |
| Other | 14 (5.9) | 5 (6.8) |  |  |
| **Resection status** |  |  | 0.722 | 0.028 |
| R0 | 216 (90.4) | 65 (87.8) |  |  |
| R1 | 17 (7.1) | 7 (9.5) |  |  |
| Rx | 6 (2.5) | 2 (2.7) |  |  |
| **UICC Stage** |  |  | 0.060 | 0.355 |
| I | 66 (27.6) | 18 (24.3) |  |  |
| II | 32 (13.4) | 18 (24.3) |  |  |
| III | 104 (43.5) | 23 (31.1) |  |  |
| IV | 37 (15.5) | 15 (20.3) |  |  |
| **Neoadjuvant therapy** |  |  | 0.007 | 0.465 |
| None | 81 (33.9) | 16 (21.6) |  |  |
| Chemotherapy | 89 (37.2) | 21 (28.4) |  |  |
| Radiochemotherapy | 68 (28.5) | 37 (50.0) |  |  |
| Radiotherapy | 1 (0.4) | 0 (0.0) |  |  |
| **Adjuvant treatment** |  |  | 0.049 | 0.368 |
| None | 187 (78.2) | 67 (90.5) |  |  |
| Chemotherapy | 47 (19.7) | 7 (9.5) |  |  |
| Radiochemotherapy | 5 (2.1) | 0 (0.0) |  |  |
| **Surgical resection** |  |  | <0.001 | 0.596 |
| Esophagectomy | 134 (56.1) | 61 (82.4) |  |  |
| Gastrectomy | 105 (43.9) | 13 (17.6) |  |  |
| **Surgical approach** |  |  | 0.082 | 0.297 |
| Open | 160 (66.9) | 45 (60.8) |  |  |
| Hybrid | 29 (12.1) | 5 (6.8) |  |  |
| MIS | 50 (20.9) | 24 (32.4) |  |  |

n (%), chi square test, *median (IQR), ^Mann Whitney U Test

(AL - anastomotic leakage, ASA - American Society of Anesthesiologists, BMI - body mass index, IQR- interquartile range, MIS - minimally invasive surgery, SMD - standardized mean difference, UICC - Union for International Cancer Control)

**Supplementary Table 2**. Type and rate of recurrence of overall cohort

|  | **AL- (n= 239)** | **AL+ (n= 74)** | **p-value (FG)** | **HR** | **95% CI** | **p-value (CSC)** |
| --- | --- | --- | --- | --- | --- | --- |
| **Recurrence (overall)** | 94 (39.3) | 32 (43.2) | 0.642 | 1.161 | 0.777–1.734 | 0.467 |
| **Lymphatic** | 40 (16.7) | 15 (20.3) | 0.508 | 1.344 | 0.743–2.434 | 0.328 |
| regional | 28 (11.7) | 11 (14.9) | 0.490 | 1.370 | 0.682–2.752 | 0.376 |
| distant | 20 (8.4) | 7 (9.5) | 0.788 | 1.239 | 0.524–2.931 | 0.626 |
| **Hematogenous** | 61 (25.5) | 25 (33.8) | 0.178 | 1.462 | 0.918–2.329 | 0.110 |
| **Peritoneal** | 33 (13.8) | 6 (8.1) | 0.219 | 0.653 | 0.274–1.558 | 0.337 |
| **Local recurrence** | 21 (8.8) | 5 (6.8) | 0.592 | 0.854 | 0.322–2.266 | 0.752 |

n (%), Fine-Grey model, cause-specific Cox regression

(AL - anastomotic leakage, CI – confidence interval, CSC – cause-specific Cox, FG – Fine-Grey, HR – hazard ratio)

**Supplementary Table 3**. Overall Survival and 1- to 5-year survival rates of overall cohort

|  | **AL- (n=230)** | **AL+ (n=67)** | **p Value** | **HR** | **95% CI** |
| --- | --- | --- | --- | --- | --- |
| **Overall survival [M]** | 37.4 | 24.7 | 0.122^ | 1.286 | 0.934–1.771 |
| **1 year** | 178 (74.5) | 49 (66.2) | 0.181 | 1.286 | 0.934–1.771 |
| **2 years** | 143 (59.8) | 37 (50.0) | 0.141 | 1.286 | 0.934–1.771 |
| **3 years** | 121 (50.6) | 33 (44.6) | 0.425 | 1.286 | 0.934–1.771 |
| **4 years** | 108 (45.2) | 28 (37.8) | 0.285 | 1.286 | 0.934–1.771 |
| **5 years** | 102 (42.7) | 24 (32.4) | 0.261 | 1.286 | 0.934–1.771 |

n (%), Fisher´s exact test, *median (IQR), ^Cox regression

(AL – Anastomotic leakage, CI – confidence interval, HR – hazard ratio, IQR - interquartile range, M - month)

**Supplementary Table 4**. Type and rate of recurrence of esophagectomy sub-cohort.

|  | **AL- (n= 84)** | **AL+ (n= 58)** | **p-value (FG)** | **HR** | **95% CI** | **p-value (CSC)** |
| --- | --- | --- | --- | --- | --- | --- |
| **Recurrence (overall)** | 33 (39.3) | 24 (41.4) | 0.887 | 1.071 | 0.580–1.980 | 0.826 |
| **Lymphatic** | 15 (17.9) | 12 (20.7) | 0.642 | 1.390 | 0.661–2.920 | 0.385 |
| regional | 8 (9.5) | 5 (8.6) | 0.858 | 1.052 | 0.310–3.573 | 0.936 |
| distant | 9 (10.7) | 8 (13.8) | 0.563 | 1.502 | 0.578–3.903 | 0.404 |
| **Hematogenous** | 22 (26.2) | 21 (36.2) | 0.171 | 1.618 | 0.925–2.830 | 0.092 |
| **Peritoneal** | 8 (9.5) | 2 (3.4) | 0.187 | 0.381 | 0.078–1.866 | 0.234 |
| **Local recurrence** | 7 (8.3) | 2 (3.4) | 0.278 | 0.469 | 0.091–2.415 | 0.365 |

n (%), Fine-Grey model, cause-specific Cox regression

(AL - anastomotic leakage, CI – confidence interval, CSC – cause-specific Cox, FG – Fine-Grey, HR – hazard ratio)

| Model | subgroup | HR | 95%CI | p value | p interaction |
| --- | --- | --- | --- | --- | --- |
| OS | AC | 1,429 | 0.842–2.425 | 0.186 | 0.774 |
| **OS** | **SCC** | **2,191** | **1.092–4.396** | **0.027** | 0.774 |
| OS | No neoadjuvant treatment | 1,028 | 0.396–2.669 | 0.955 | 0.496 |
| **OS** | **Neoadjuvant treatment** | **1,633** | **1.063–2.509** | **0.025** | 0.496 |
| **OS** | **Esophagectomy** | **1,633** | **1.045–2.551** | **0.031** | 0.527 |
| OS | Gastrectomy | 2,822 | 0.371–21.482 | 0.316 | 0.527 |
| RFS | AC | 1,079 | 0.580–2.007 | 0.810 | 0.723 |
| RFS | SCC | 1,673 | 0.806–3.472 | 0.167 | 0.723 |
| RFS | No neoadjuvant treatment | 0,749 | 0.256–2.197 | 0.599 | 0.253 |
| RFS | Neoadjuvant treatment | 1,376 | 0.856–2.212 | 0.187 | 0.253 |
| RFS | Esophagectomy | 1,298 | 0.799–2.109 | 0.292 | 0.329 |
| RFS | Gastrectomy | 1,211E-12 | 0-n.a. | 0.997 | 0.329 |

**Supplementary Table 5**. Subgroup analysis of AL+ for OS and RFS.

Cox regression

(AC – adenocarcinoma, AL - anastomotic leakage, CI – confidence interval, HR – hazard ratio, OS - overall survival, RFS - recurrence-free survival,SCC – squamous cell carcinoma)

# Figures

**Supplementary Figure 1**. Love Plot (A) and Common Support (B)

A


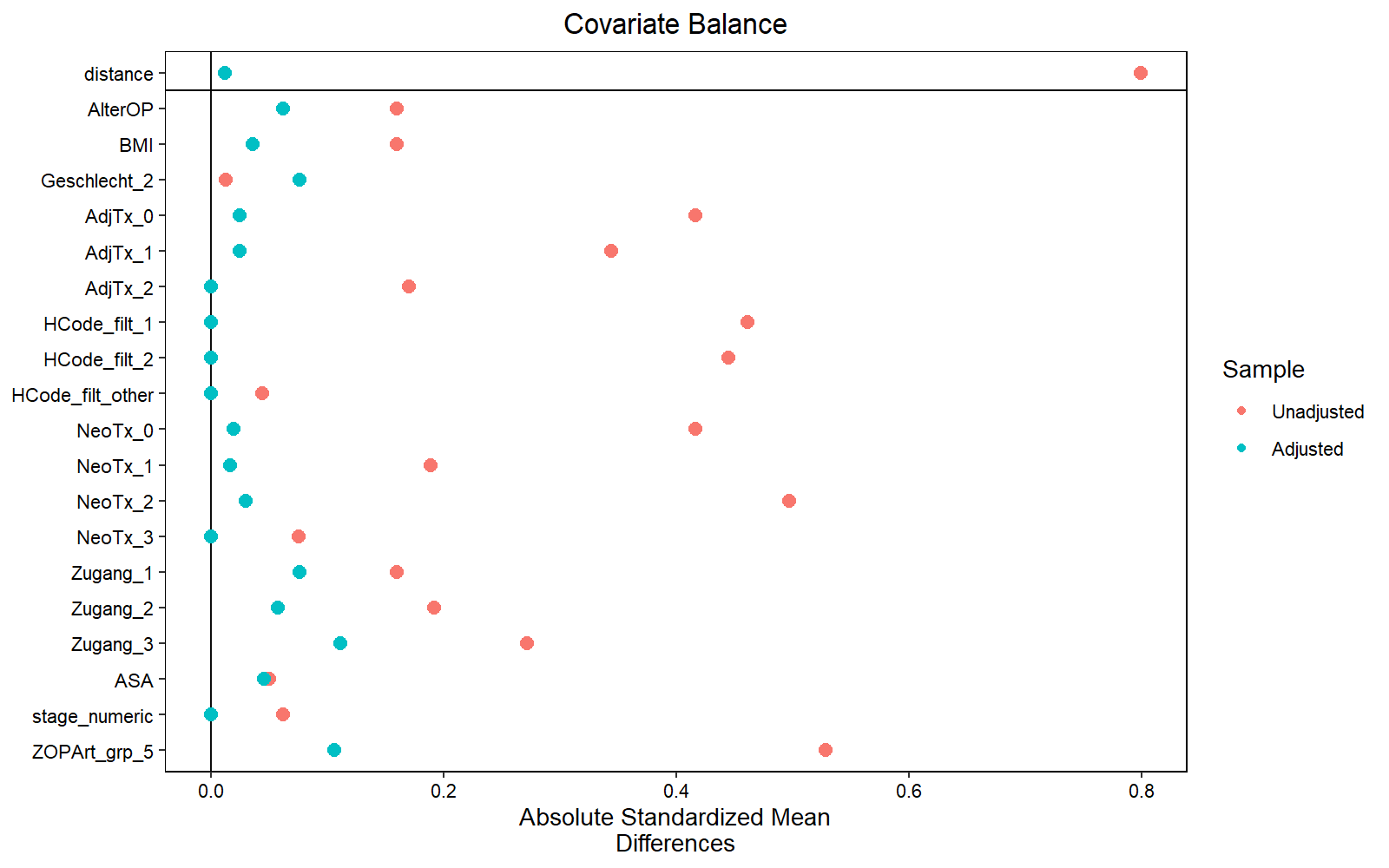


B
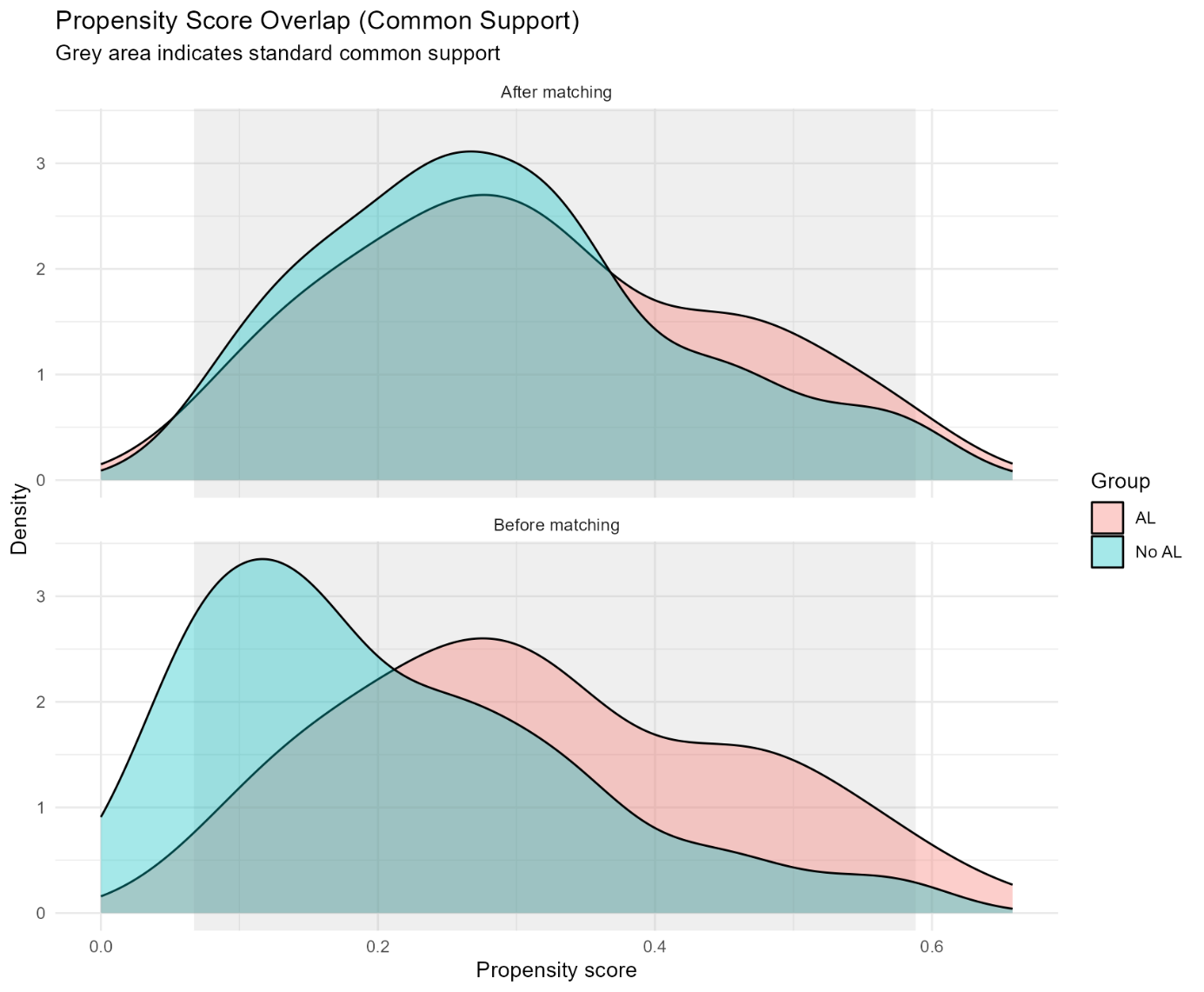


**Supplementary Figure 2**. OS (A) and RFS (B) depending on the ECCG severity grade of AL.

A


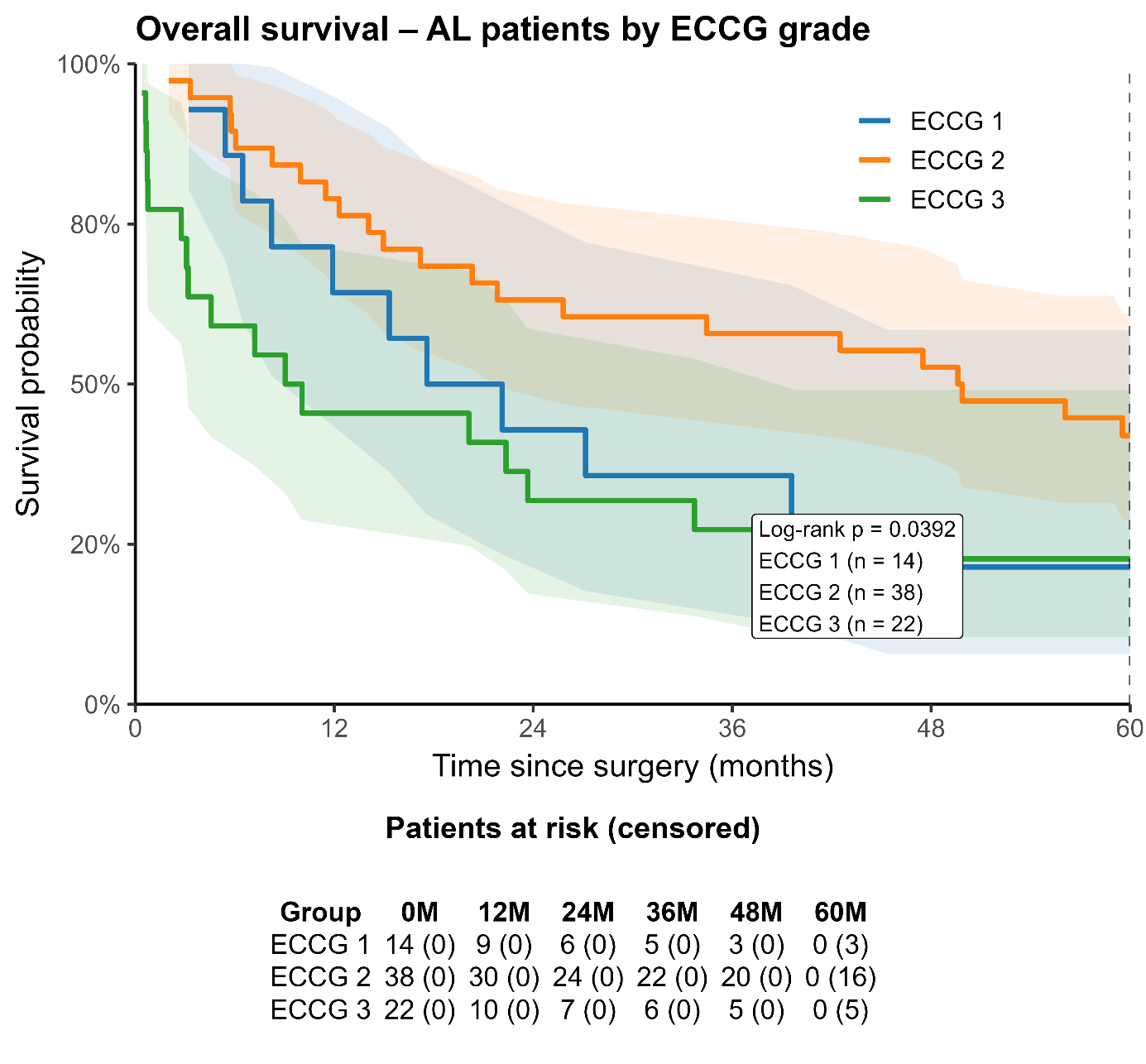


B
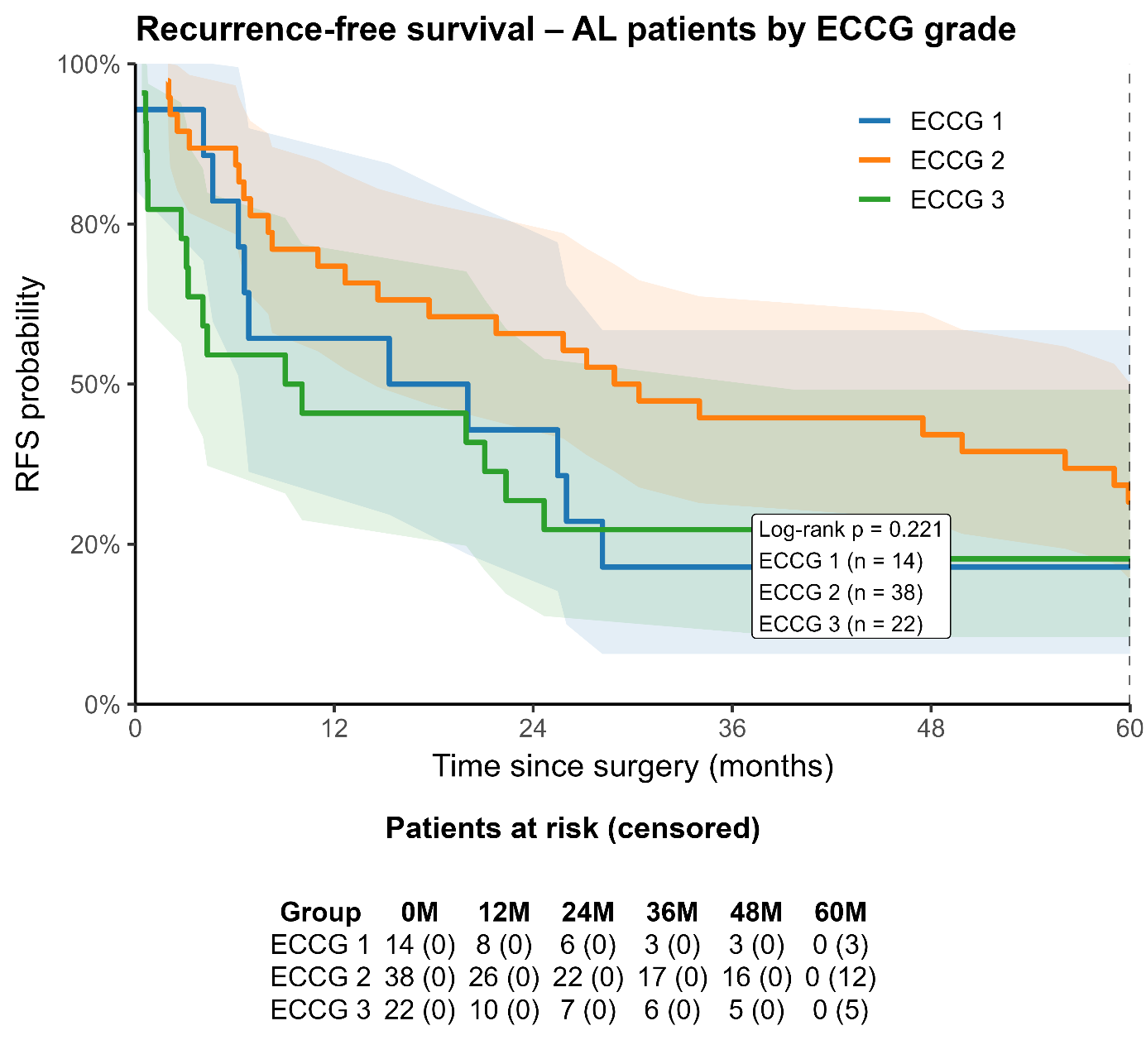


**Supplementary Figure 3**. OS (A) and RFS (B) in the esophagectomy sub-cohort.

A
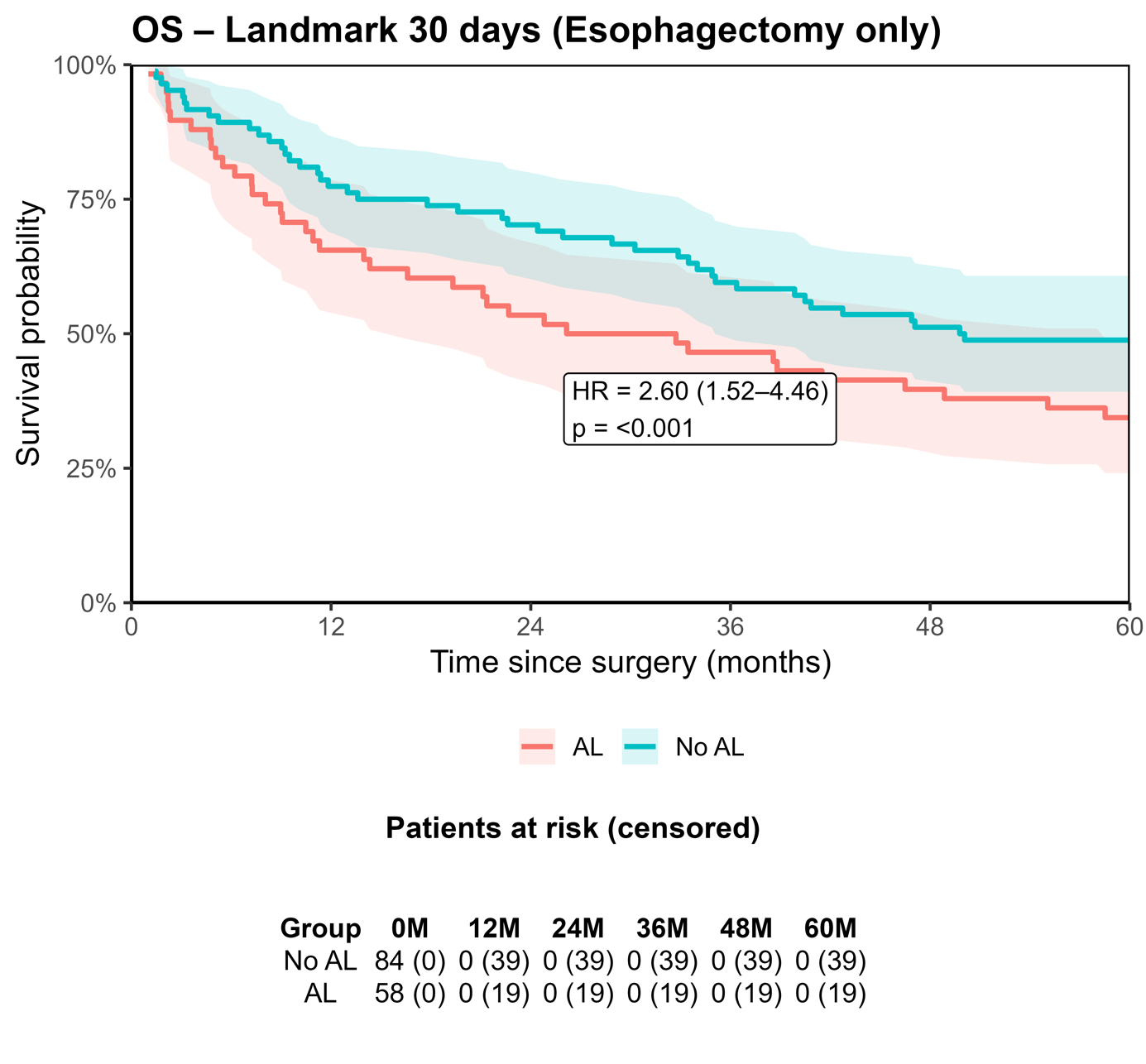


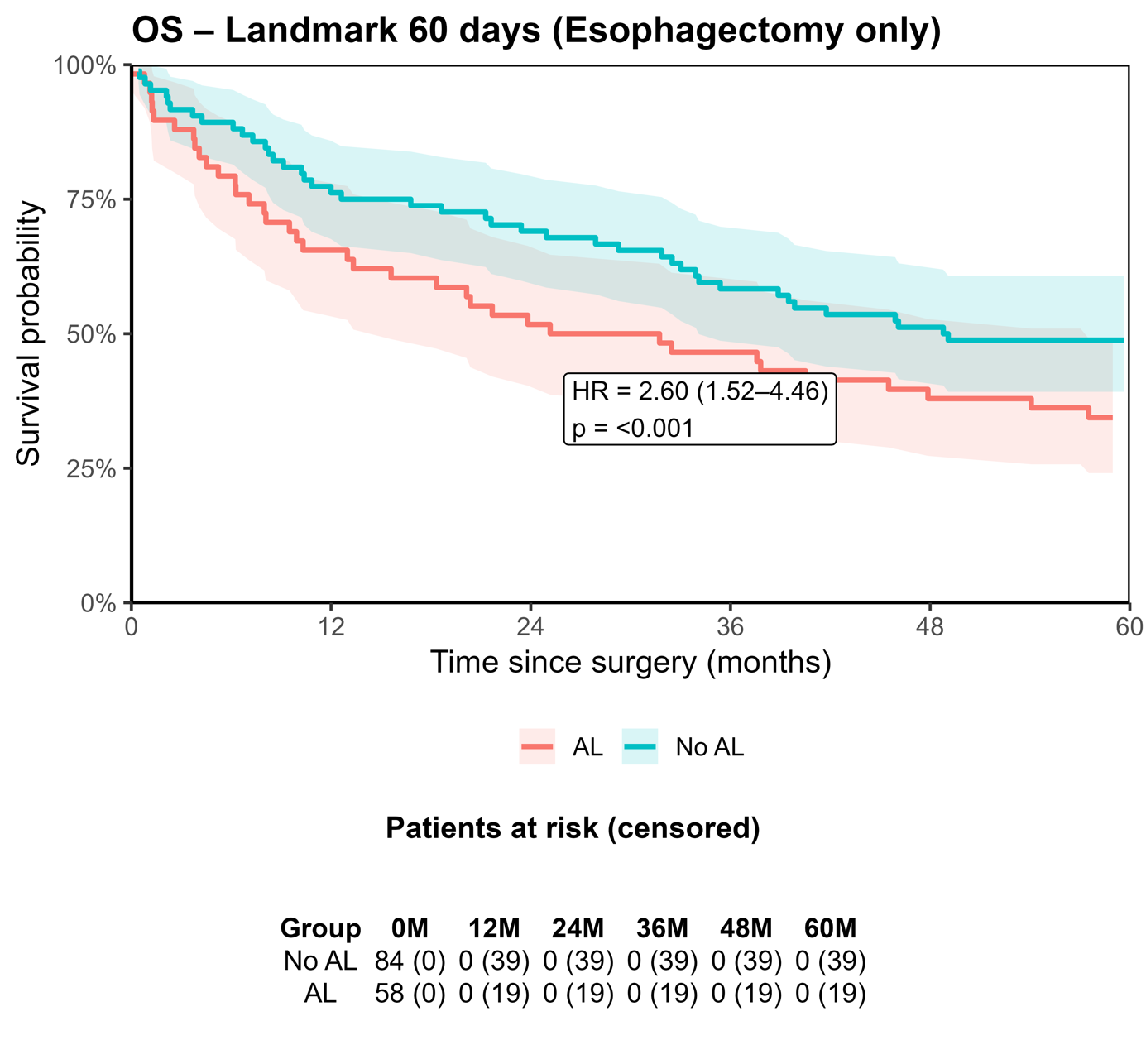


B
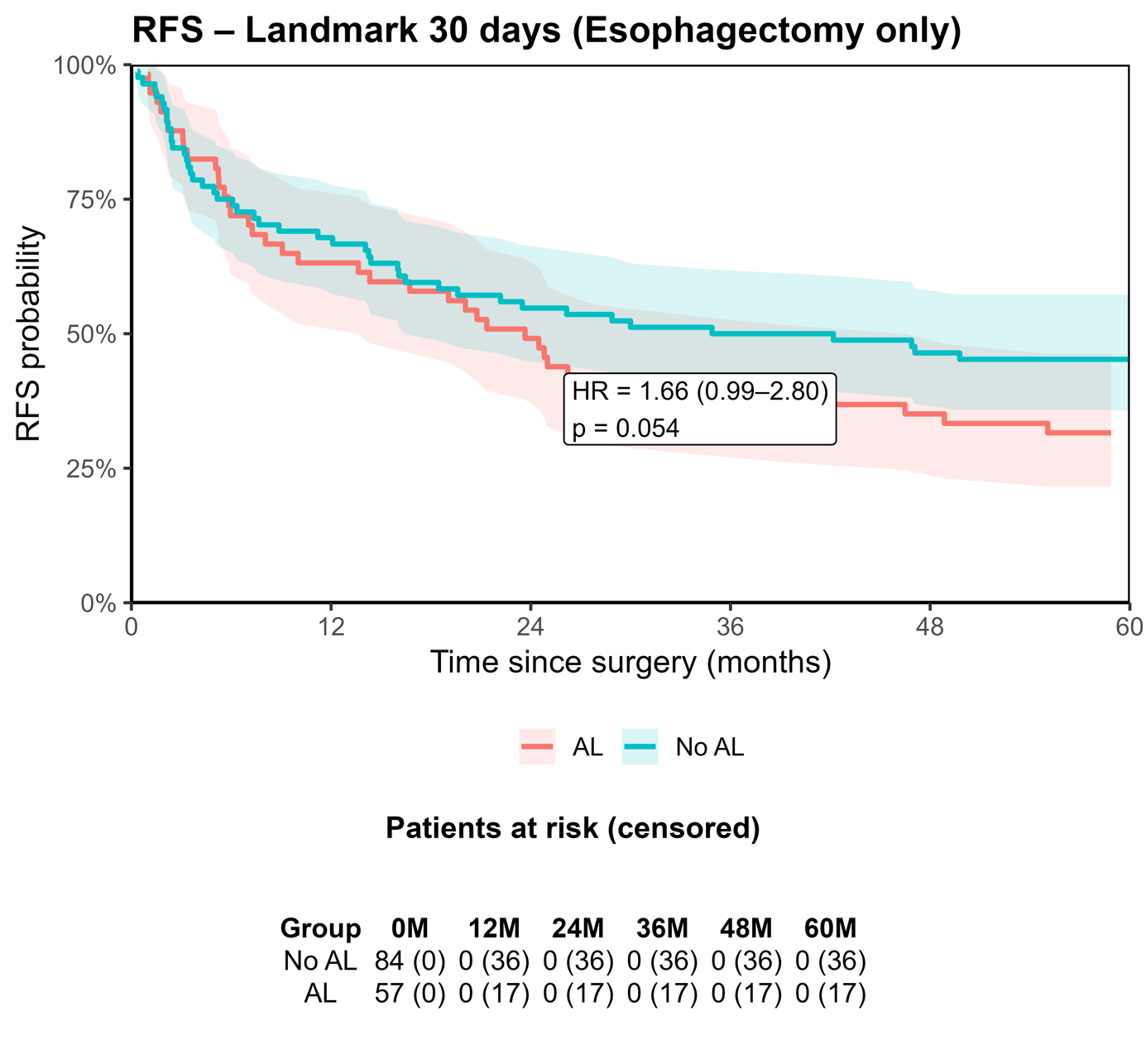


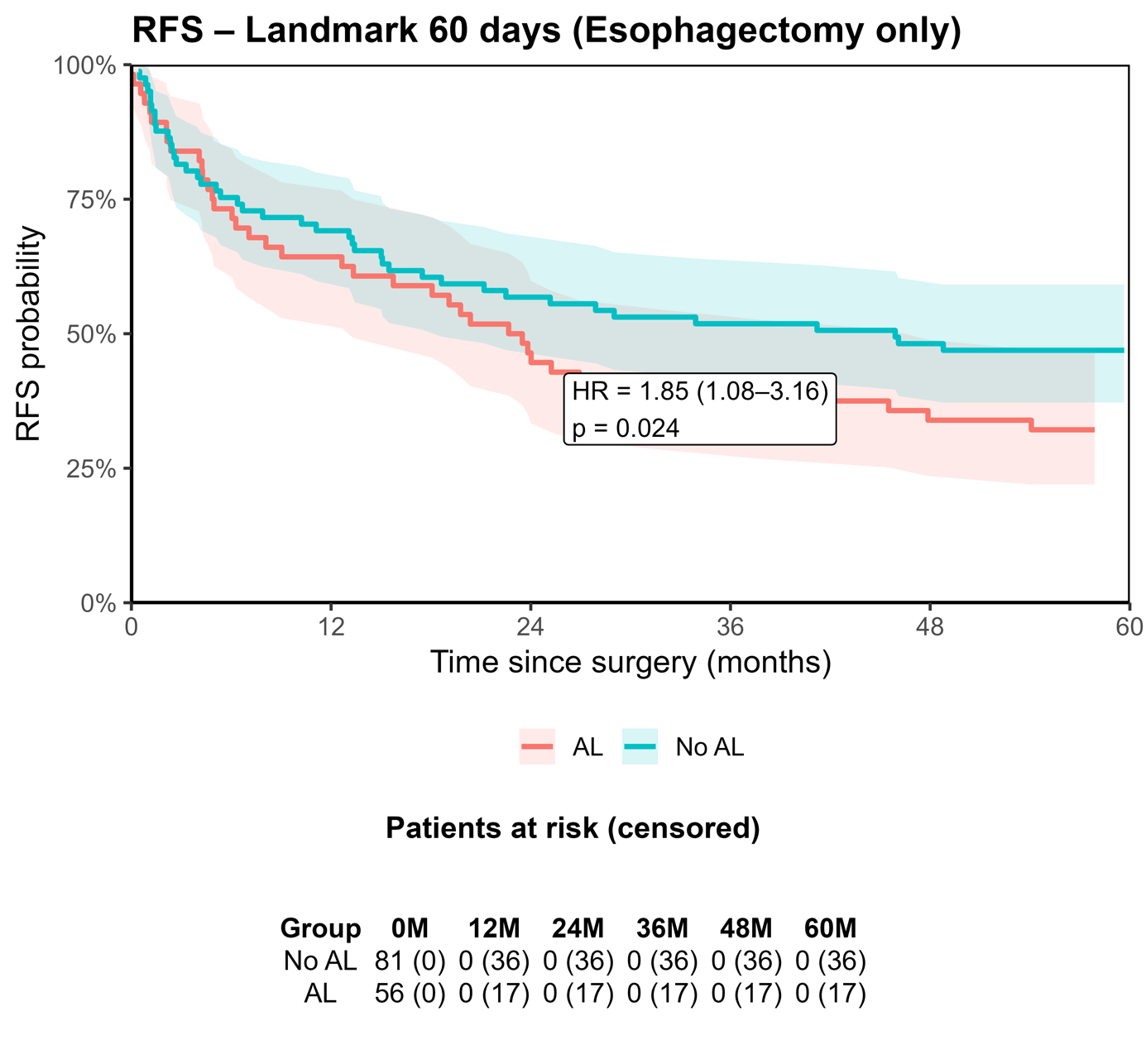

Supplement: Supplementary file 1 — Supplementary Material 1. [file 12893_2026_3681_MOESM1_ESM.docx]
